# Polymer Analysis

**DOI:** 10.3390/polym12010052

**Published:** 2019-12-31

**Authors:** Giulio Malucelli

**Affiliations:** Department of Applied Science and Technology, and local INSTM Unit, Viale Teresa Michel 5, 15121 Alessandria, Italy; giulio.malucelli@polito.it

Dear colleagues and friends,

The year 2019 is almost at an end, and our Polymer Analysis section has experienced another fruitful year of high-quality Special Issues and scientific papers devoted to different topics of polymer analysis.

In particular, some of them thoroughly investigated new insights on flame retardance of biopolymer composites (Investigation of the Flammability and Thermal Stability of Halogen-Free Intumescent System in Biopolymer Composites Containing Biobased Carbonization Agent and Mechanism of Their Char Formation [1]), polyurethane composites (Preparation and Flame Retardance of Polyurethane Composites Containing Microencapsulated Melamine Polyphosphate [2]), glass fiber-reinforced polybutylene terephthalate composites (Development of a Semiglobal Reaction Mechanism for the Thermal Decomposition of a Polymer Containing Reactive Flame Retardants: Application to Glass-Fiber-Reinforced Polybutylene Terephthalate Blended with Aluminum Diethyl Phosphinate and Melamine Polyphosphate [3]) carbon fiber-reinforced composites (Flame Retardancy of Low-Viscosity Epoxy Resins and Their Carbon Fibre Reinforced Composites via a Combined Solid and Gas Phase Mechanism [4]), and rigid polyurethane foams (Fire Phenomena of Rigid Polyurethane Foams [5]). 

Furthermore, special attention was devoted to the design, preparation, and characterization of polymer micro- and nanocomposites containing modified graphene oxide (Octadecylamine-Grafted Graphene Oxide Helps the Dispersion of Carbon Nanotubes in Ethylene Vinyl Acetate [6]), hydrotalcite (Ionic Liquid as Surfactant Agent of Hydrotalcite: Influence on the Final Properties of Polycaprolactone Matrix [7]), graphene oxide (Toughening of Poly(lactic acid) and Thermoplastic Cassava Starch Reactive Blends Using Graphene Nanoplatelets [8]), graphite and carbon black (PLA Melt Stabilization by High-Surface-Area Graphite and Carbon Black [9]), and polyhedral oligomeric silsesquioxane (POSS) (Minimizing the Strong Screening Effect of Polyhedral Oligomeric Silsesquioxane Nanoparticles in Hydrogen-Bonded Random Copolymers [10]).

Significant developments were also achieved in the characterization of crystallization phenomena taking place in polymers and polymer composites (How Chain Intermixing Dictates the Polymorphism of PVDF in Poly(vinylidene fluoride)/Polymethylmethacrylate Binary System during Recrystallization: A Comparative Study on Core–Shell Particles and Latex Blend [11]; Development of Crystalline Morphology and Its Relationship with Mechanical Properties of PP/PET Microfibrillar Composites Containing POE and POE-g-MA [12]) and of phase transitions in polymer and copolymer systems as well (Deformation-Induced Phase Transitions in iPP Polymorphs [13]; Polymorphic Behavior and Phase Transition of Poly(1-Butene) and Its Copolymers [14]). 

Finally, a very challenging and up-to-date paper assessed the suitability of additive manufacturing (namely, fused deposition modelling (FDM)) for tissue engineering applications (Independent Evaluation of Medical-Grade Bioresorbable Filaments for Fused Deposition Modelling/Fused Filament Fabrication of Tissue Engineered Constructs [15]).

All these few examples clearly witness the activity in the Polymer Analysis section of *Polymers*. I would remind all of you that 15 Special Issues are still open and ready to receive your valuable manuscripts.

I hope that in 2020 the Polymer Analysis section will continue to grow and expand with the help of all the Editorial Board, the Editorial Staff, and (last but not least) all the readers and authors!
